# Amniotic membrane and/or umbilical cord tissue for treatment of facet joint syndrome: a narrative review

**DOI:** 10.1186/s13018-023-04241-2

**Published:** 2023-10-02

**Authors:** Ashim Gupta, Nicola Maffulli

**Affiliations:** 1 Regenerative Orthopaedics, Noida, India; 2 Future Biologics, Lawrenceville, GA USA; 3 BioIntegrate, Lawrenceville, GA USA; 4 South Texas Orthopaedic Research Institute (STORI Inc.), Laredo, TX USA; 5 Department of Musculoskeletal Disorders, School of Medicine and Surgery, University of Salerno, Fisciano, Italy; 6 San Giovanni di Dio e Ruggi D’Aragona Hospital “Clinica Ortopedica” Department, Hospital of Salerno, Salerno, Italy; 7 Barts and the London School of Medicine and Dentistry, Centre for Sports and Exercise Medicine, Queen Mary University of London, London, UK; 8 School of Pharmacy and Bioengineering, Keele University School of Medicine, Stoke On Trent, UK

**Keywords:** Amniotic membrane, Umbilical cord, Perinatal tissue, Regenerative medicine, Facet joint syndrome, Spondylosis, Chronic back pain, Low back pain

## Abstract

Musculoskeletal spine disorders, especially low back pain, induce enormous amounts of stress and financial burden on individuals and healthcare systems throughout the world. Disorders of the facet joints in the lumbar spine are the most predominant cause of back pain, resulting in facet joint syndrome (FJS). Conventional treatments for FJS are short-lived and have limitations and side effects. Thus, safer and more effective alternatives that can reduce pain and improve patient-reported outcomes are needed. Recently, the utilization of biologics, including the ones derived from perinatal tissue such as amniotic membrane (AM) and umbilical cord (UC), has significantly increased for regenerative medicine applications. This manuscript summarizes the outcomes of preclinical and clinical studies utilizing AM and/or UC for FJS. We identified no preclinical studies and 3 retrospective studies utilizing the search terms “amniotic membrane” and/or “umbilical cord” and “facet joint syndrome”. The administration of AM + UC is safe and potentially efficacious for patients with FJS. However, more preclinical studies and appropriately powered, multi-center, prospective non-randomized and randomized controlled studies with longer follow-up are warranted to further evaluate the efficacy of AM + UC to justify its clinical use.

## Introduction

Musculoskeletal disorders are a leading cause of disability and significantly impact physical activity and sleep quality, inducing depression and cognitive impairment [1, 2]. Chronic low back pain, the most common musculoskeletal issue, is the foremost reason of disability, affecting millions of people per year throughout the world [3, 4]. Back pain arises for many causes, and the facet joints in the lumbar spine account for 15–45% of all cases of low back pain [5]. The facet joint comprises of a synovial capsule, synovial membrane, cartilage (hyaline) and sub-chondral bone responsible for compression/tension resistance and mobility [6, 7]. Spinal joints may degenerate over time because of release of inflammatory cytokines as a result of natural wear and tear or abnormal body mechanics, stemming from repetitive stress or repeated long-term low-level trauma, leading to facet joint syndrome (FJS) or spondylosis [5, 8–10].

Current conservative multimodal treatment for FJS consists of activity modification, physical therapy, non-steroidal anti-inflammatory drugs (NSAIDs), non-narcotic and narcotic analgesics, and steroids [11–13]. These conventional options induce short-lived amelioration of symptoms and have side effects [14]. Hence, there is a need for safer and more effective alternatives for FJS treatment. Recently, there has been a marked increase in the use of biologics for regenerative medicine applications, including chronic musculoskeletal pain conditions. These biologics are derived from both autologous (e.g., platelet-rich plasma, bone marrow concentrate, adipose tissue) and allogenic perinatal tissues. Specifically, perinatal tissues including amniotic membrane (AM) and umbilical cord (UC) are widely studied because of commercial availability and their demonstrated safety and efficacy in patients with musculoskeletal conditions given their anti-scarring, anti-inflammatory and pro-regenerative properties [14–22]. Thus far, only limited studies have investigated the safety and efficacy of amniotic membrane and/or umbilical cord tissue for treatment of FJS. The primary objective of this study is to summarize the outcomes of preclinical and clinical studies using AM and/or UC tissue and associated mesenchymal stem cells in patients with FJS. The secondary objective is to document the ongoing clinical trials registered on clinicaltrials.gov on AM and/or UC tissue for management of FJS.

## Materials and methods

### Search criteria

**Fig. 1:**
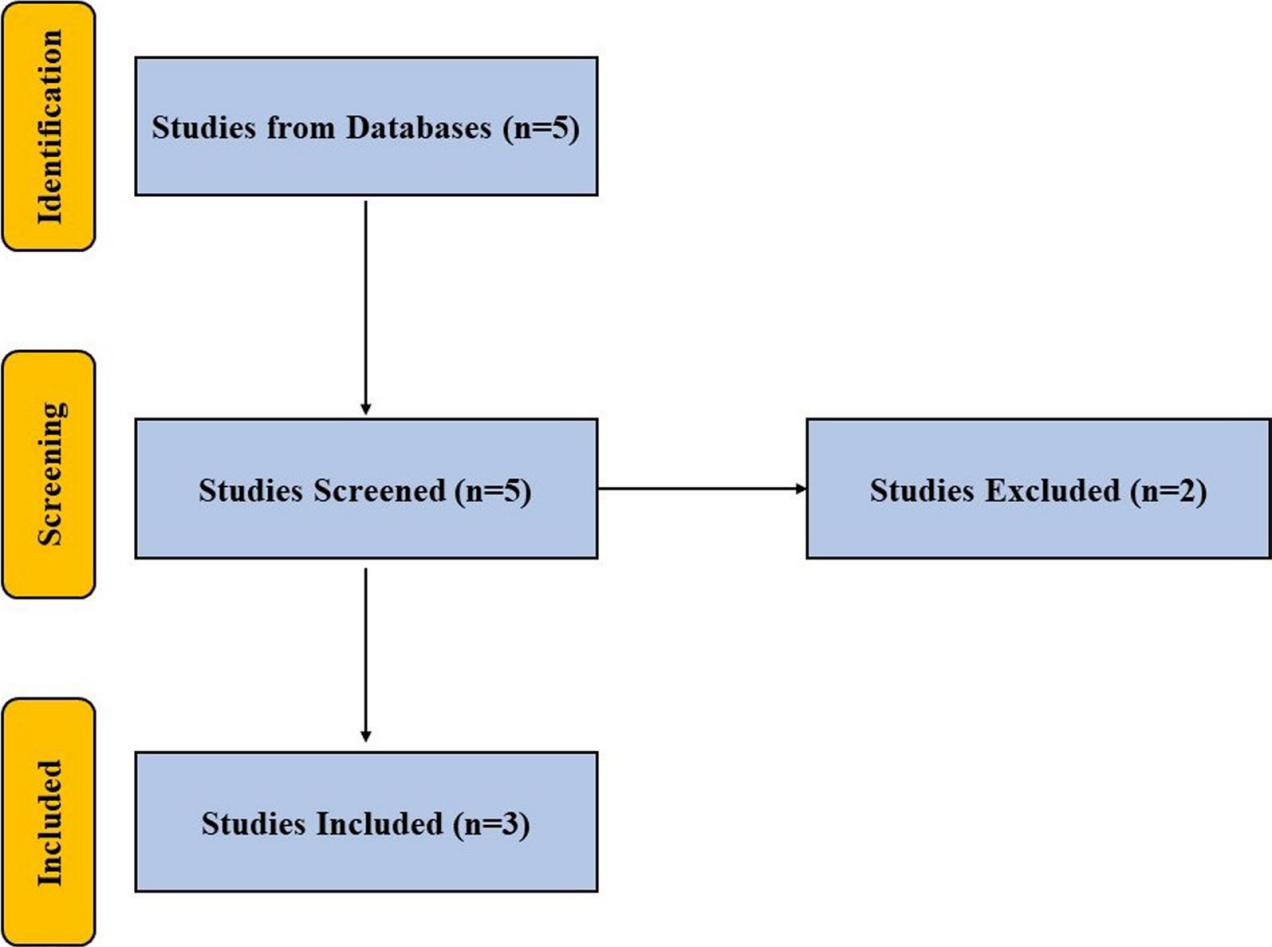
Flow diagram

A search was performed using the terms “amniotic membrane” and/or “umbilical cord” and “facet joint syndrome” in PubMed, ScienceDirect and Google Scholar databases for articles published in English to May 28, 2023. All preclinical and clinical studies utilizing AM and/or UC for facet joints were included in this manuscript. Studies not using AM and/or UC or not focusing on management of FJS were excluded. A flow diagram is shown in Fig. 1.

In addition, we searched ClinicalTrials.gov using the same search terms to identify registered trials on the use of AM and/or UC for the management of FJS.

## Results

### Pre-clinical studies

To date, no preclinical studies regarding the use of amniotic membrane and/or umbilical cord tissue for the treatment of FJS have been published.

### Clinical studies

A retrospective chart review assessed the safety and effectiveness of intra-articular AM + UC in providing symptomatic relief in patients with FJS [23]. Patients 18–85 years old with at least 6 months of follow-up data were included. Patients with a confirmed diagnosis of FJS were injected under fluoroscopic guidance with 50mg of AM + UC suspended in 0.5mL preservative free saline (0.4mL were injected in the zygapophysial intra-articular space and 0.1mL in the inferior capsule area). Patients charts were reviewed for demographics (age, gender, body mass index), diagnosis, use of opioids, and subjective pain score (scale of 0–10; 0 represents no pain and 10 represents most severe pain). Of 30 patients treated, only 9 (7 males and 2 females; average age 52.1 ± 15.9 years) met the inclusion criteria. 5 patients had cervical facet joints (trauma) and 4 patients had lumbar facet joints (1 injury and 3 osteoarthritis) affected. All patients reported severe pain, 8.2 ± 0.4 at baseline, which significantly (*p* < 0.05) reduced to 0.4 ± 0.7 (94.6% reduction) at 6 months follow-up. Additionally, all patients at presentation reported pain despite oral NSAIDs and acetaminophen, and 4 patients took opioids (> 100 MME (morphine milligram equivalents) per day). At 6 months follow-up, all patients had stopped pain medications including opioids. Moreover, no additional interventions were needed, and no adverse events were reported during the 6 months of follow-up. Despite the small sample size, this was one of the first studies demonstrating safety and efficacy of AM + UC in patients suffering with FJS.

A retrospective cohort study evaluated the safety and efficacy of AM + UC injection in managing FJS pain [24]. Patients with confirmed FJS diagnosis (confirmed via radiography, MRI and diagnostic anesthetic block testing) and who received AM + UC intra- or peri-articular injections were included in the study. Patients who received concurrent treatment for other spinal pathologies had dementia or no follow-up data were excluded. These patients received injections mainly at 3 or 4 levels of lumbar spine, corresponding to dose administration of 12.5mg to 16.7mg of AM + UC per joint. The Numeric Pain Rating Scale (NPRS; 0–10 scale, 0: no pain and 10: worst imaginable pain) score was documented at baseline, and at follow-up visits (6 weeks, and 3, 6 and 12 months post-injection), patient-reported Patient Global Impression of Change (PGIC) was recorded. 54 patients (23 males and 31 females, mean age 69.7 ± 13.4 years) met the eligibility criteria. The mean NPRS score at baseline was 9.2 ± 1.0, despite prior treatments including activity modification, physical therapy, and use of NSAIDs and opioids in 66.7%, 35.2%, 61.1% and 37.0% of patients, respectively. 70.8% (*N* = 38) of the patients showed at least 30% improvement (responders_30_, patients were classified as responders if they showed at least 30% improvement at their 1st follow-up visit) and 57.4% (*N* = 31) of the patients showed at least 50% improvement (responders_50_, patients who showed at least 50% improvement at their 1^st^ follow-up visit) in PGIC. The mean PGIC improvement was 65.3%, 67.5%, 56.9%, 56.7% for responders_30_ and 72.2%, 69.6%, 65.0%, 45.0% for responders_50_ at 6 weeks and 3, 6 and 12 months follow-up visits, respectively. The number of patients using NSAIDs decreased significantly (*p* < 0.01) at follow-up visits compared to baseline, with no change for opioid users. Additionally, no adverse events were reported during 12 months of follow-up. Despite limitations such as small sample size and the retrospective nature of the study, the results are in accordance with study by Bennett et al. [23] and demonstrate the safety and efficacy of AM + UC in patients suffering with FJS.

A retrospective chart review evaluated the safety and efficacy of AM + UC in patients with spinal musculoskeletal disorders [25]. Inclusion criteria involved patients ≥ 18 years of age, presence of spinal musculoskeletal condition such as radiculopathy, herniated disks, spinal stenosis, osteoarthritis injection of AM + UC, recording of baseline pain score, and at least one follow-up visit. The patients received either lumbar and cervical injections or both epidural and facet injections depending on the location and severity of symptoms. Some patients also received 2 injections of the same type, 4 weeks apart. The primary outcome was change in pain (using NPRS) at 2, 3, 4, 6 and 8 weeks post-injection compared to baseline. The secondary outcome measures included increased range of motion (ROM), return to full work activities, return to activities of daily living, increased sensation, decrease in radicular nerve pain, and decrease in non-narcotic and narcotic analgesics usage. A total of 52 patients (35 males and 17 females) with a mean age of 40.8 ± 9.6 years were included in the study with the main diagnosis of spondylosis in 84.6% (*n* = 44), intervertebral disk disease in 59.6% (*n* = 31), and radiculopathy in 34.6% (*n* = 18) of the patients. The pain scores significantly (*p* < 0.05) reduced at 2 weeks (3.4 ± 2.3) and at 3–4 weeks (3.5 ±) compared to baseline pain score (4.9 ± 2.2). The pain score also reduced at 6 weeks and 8 weeks, but the change was not significant (*p* > 0.05) compared to baseline. However, at a mean follow-up time of 10.6 ± 5.4 weeks, pain was significantly reduced compared to baseline (*p* < 0.0001). All patients also reported improvements in pain, ROM and sensation. No severe adverse events were reported throughout the duration of this study. The results from this study are in accordance with aforementioned studies [23, 24] and provide evidence of safety and effectiveness of AM + UC injected in the facet joints.

### On-going clinical studies

As of 28 May 2023, there are no clinical trials registered on clinicaltrials.gov (search terms, “amniotic membrane” and/or “umbilical cord” and “facet joint”) evaluating the safety and efficacy of amniotic membrane and/or umbilical cord for the treatment of facet joint syndrome.

## Discussion

The present study evaluated the therapeutic potential of AM and/or UC for the management of FJS. Preclinical and clinical studies focusing on the effect of AM and/or UC tissue and derived mesenchymal stem cells on FJS were included. Based on our search strategy and inclusion/exclusion criteria, no preclinical studies and only three clinical studies fit the scope of our study.

The treatment of chronic back pain resulting from FJS poses a great challenge to physicians [23]. The lack of an effective gold standard treatment results in significant disability for patients and considerable burden on health care systems across the globe [23]. Intra-articular injections of steroids are commonly used, but no short or long-term benefits, or benefits compared to placebo injection, were observed [23–27]. Bennett et al. [23] showed pian reduction by over 94%, much better compared to what is reported for corticosteroids at 6 months follow-up. Castellanos et al. [24] reported at least 50% pain improvement (considered clinically significant) in over 57% of the patients. This was in accordance with the study by Bennett et al. Moreover, the study by Ross et al. [25], similar to studies by Bennett et al. and Castellanos et al., showed significant reduction in pain as early as 2 weeks post-injection. The results from these preliminary studies are consistent with other studies reporting benefits of AM and/or UC for various musculoskeletal injuries [25, 28]. There are no on-going clinical trials registered on clinicaltrials.gov.

## Conclusion

Despite constraints, including lack of preclinical and prospective clinical studies, the aforementioned retrospective studies show that administration of AM + UC is safe and potentially efficacious in patients with FJS. However, more in vitro and preclinical studies are warranted to determine the mechanism of action of AM and/or UC tissue and associated mesenchymal stem cells in managing FJS. Additionally, appropriately powered, multi-center, prospective non-randomized and randomized controlled studies with longer follow-up are required to further assess the efficacy of AM + UC and ultimately justify its clinical use in FJS patients.

## Data Availability

All relevant data are contained within the article.

## Abbreviations

FJS: Facet joint syndrome
AM: Amniotic membrane
UC: Umbilical cord
NSAIDs: Non-steroidal anti-inflammatory drugs
MME: Morphine milligram equivalents
MRI: Magnetic resonance imaging
NPRS: Numeric pain rating scale
PGIC: Patient global impression of change
ROM: Range of motion
